# Combining Molecular Weight Fractionation and Metabolomics to Elucidate the Bioactivity of Vegetal Protein Hydrolysates in Tomato Plants

**DOI:** 10.3389/fpls.2020.00976

**Published:** 2020-06-30

**Authors:** Luigi Lucini, Begoña Miras-Moreno, Youssef Rouphael, Mariateresa Cardarelli, Giuseppe Colla

**Affiliations:** ^1^Department for Sustainable Food Process, Research Centre for Nutrigenomics and Proteomics, Università Cattolica del Sacro Cuore, Piacenza, Italy; ^2^Council for Agricultural Research and Economics—Research Centre for Genomics and Bioinformatics (CREA-GB), Fiorenzuola d'Arda, Italy; ^3^Department of Agricultural Sciences, University of Naples Federico II, Portici, Italy; ^4^Consiglio per la ricerca in agricoltura e l'analisi dell'economia agraria, Centro di ricerca Orticoltura e Florovivaismo, Pontecagnano Faiano, Italy; ^5^Department of Agriculture and Forest Sciences, University of Tuscia, Viterbo, Italy

**Keywords:** dialysis fractionation, rooting assay, auxin-like activity, plant secondary metabolism, *Solanum lycopersicum* L.

## Abstract

The comprehension of the bioactive fractions involved in the biostimulant activity of plant derived protein hydrolysates (PH) is a complex task, but it can also lead to significant improvements in the production of more effective plant biostimulants. The aim of this work is to shed light onto the bioactivity of different PH dialysis fractions (PH1 < 0.5–1 kDa; PH2 > 0.5–1 kDa; PH3 < 8–10 kDa; PH4 > 8–10 kDa) of a commercial PH-based biostimulant through a combined *in vivo* bioassay and metabolomics approach. A first tomato rooting bioassay investigated the auxin-like activity of PH and its fractions, each of them at three nitrogen levels (3, 30, and 300 mg L^−1^ of N) in comparison with a negative control (water) and a positive control (indole-3-butyric acid, IBA). Thereafter, a second experiment was carried out where metabolomics was applied to elucidate the biochemical changes imposed by the PH and its best performing fraction (both at 300 mg L^−1^ of N) in comparison to water and IBA. Overall, both the PH and its fractions increased the root length of tomato cuttings, compared to negative control. Moreover, the highest root length was obtained in the treatment PH1 following foliar application. Metabolomics allowed highlighting a response to PH1 that involved changes at phytohormones and secondary metabolite level. Notably, such metabolic reprogramming supported the effect on rooting of tomato cuttings, being shared with the response induced by the positive control IBA. Taken together, the outcome of *in vivo* assays and metabolomics indicate an auxin-like activity of the selected PH1 fraction.

## Introduction

Nowadays, the improvement of sustainable agronomic practices to reduce the input of chemical inputs and to improve environmental aspects and quality of agricultural productions is becoming mandatory (Searchinger et al., 2013; De Pascale et al., 2017). With this regard, several technological innovations have been proposed, including the use of bio-based products such as plant biostimulants (Rouphael and Colla, 2018; Rouphael et al., 2018b; Xu and Geelen, 2018). Plant biostimulants include substances and/or microorganisms that are able to enhance plant growth, tolerance to abiotic stress, water, and nutrients use efficiency, rather than promote nutritional and functional quality of the products (Du Jardin, 2015; Rouphael et al., 2018b). Different classes of non-microbial and microbial products have been proposed among biostimulants, such as beneficial microorganisms (e.g. mycorrhiza or plant growth promoting rhizobacteria), humic substances, seaweed extracts, and protein hydrolysates (PH) (Calvo et al., 2014; Battacharyya et al., 2015; Canellas et al., 2015; Colla et al., 2015; Haplern et al., 2015; Rouphael et al., 2015; Ruzzi and Aroca, 2015; Colla et al., 2017a; Bitterlich et al., 2018). Among organic non-microbial plant biostimulants, humic acids and PH command half of the market share (Rouphael and Colla, 2018). These latter are a mixture of peptides and free amino acids resulting from the chemical or enzymatic partial hydrolysis of protein sources from either animal or vegetal origin (Colla et al., 2015; Colla et al., 2017b). The applications of PH-based biostimulants have been reported to enhance nutrient use efficiency and tolerance to abiotic stressors such as drought, extreme temperatures, and salinity (Calvo et al., 2014; Haplern et al., 2015; Lucini et al., 2015; Colla et al., 2017a; Colla et al., 2017b; Rouphael et al., 2017a; Rouphael et al., 2017b; Carillo et al., 2019a; Carillo et al., 2019b). In a recent review Colla and co-workers were able to summarize the main physiological and molecular mechanisms behind the biostimulant action of PH (Colla et al., 2017a). Direct and indirect mechanisms include: i) stimulation the C and N metabolism by triggering key enzymes, ii) increasing the antioxidant defense systems, iii) triggering hormone-like activities, and modulating the root system apparatus thus increasing nutrient uptake/assimilation and consequently boosting crop productivity.

Considering that several molecular mechanisms and biochemical processes have been related to PH activity to crops, it is clear that the biostimulant action is far beyond a mere supply of amino acids as nitrogen source. Besides representing an available source of nitrogen and carbon skeletons, the peptides in PH are supposed to exert a direct regulatory activity toward plants growth, known as hormone-like activity (Colla et al., 2014; Oh et al., 2018; Tejada et al., 2018). Signaling peptides are mainly short-amino acid chains (2–50 amino acids), having specific amino acid primary sequences and inducing biological effects at very low concentration (nM). Matsumiya and Kubo (2011) identified in PH obtained through enzymatic hydrolysis of soybean meal a signaling peptide having a 12 amino acid sequence; this peptide, the so-called “root hair promoting peptide”, seems to stimulate key gene(s) that increase root number and length of root hair. Moreover, some amino acids can also exert a signaling role. As an example, L-glutamate was shown to inhibit primary root growth and increase root branching near the root apex when roots were exposed to low concentrations of the amino acid (Forde and Lea, 2007). Noteworthy, the information on the substances being actually responsible of the biostimulant activity of PH is still limited, and a synergic role of different components has been recently postulated (Paul et al., 2019a; Paul et al., 2019b). Nonetheless, different contributions of PH fractions can be postulated, with small molecules (including amino acids), oligopeptides, and polypeptides likely representing the fractions having a potential biostimulant activity.

The comprehension of the bioactive substances involved in the biostimulant activity is a complex task, but it can also lead to significant improvements in the field of plant biostimulants. Indeed, the comprehension of the components to which biological activity is related can assist the choice of the best sources for PH, the improvement of hydrolysis processes and PH manufacture in general and can support the definition of the best agronomic strategies. Taken together, these improvements might open the field toward new generation biostimulants (2.0). Given the complex composition of products from natural origin such as biostimulants, the understanding of the most active components can be achieved following fractionation through molecular weight cut-off. The results can assist companies to optimize the production process in order to maximize the amount of the most active fraction(s).

Taking into account that a PH is composed by several components differing for chemical structure and molecular weight, and considering that no information is available on the actual contribution of each specific fraction, the aim of this work is to shed light onto the biostimulant activity of the different components of Trainer^®^, a representative commercially available PH. With this purpose, molecular fractionation, *in vivo* bioassays and plant metabolomics were combined to investigate the contribution of low-molecular-weight components such as amino acids and peptides on the activity and mode(s) of action of a PH. Because the whole PH product and its fractions were tested up to doses corresponding to 300 mg of N L^−1^, we cannot exclude that such high doses of PH and its fractions may act not only as biostimulants but also as sources of nitrogen. Besides this specific case, the approach proposed might find application in all cases where a biostimulant product is composed by a mixture of small molecules and high molecular weight macro-biomolecules.

## Materials and Methods

### The PH and Its Fractionation

The legume-derived PH biostimulant Trainer^®^ was a commercial product manufactured by Italpollina (Rivoli Veronese, Italy), and purchased from a commercial retailer. This PH is obtained by hydrolysis of proteins derived from legume seed ﬂour that underwent enzymatic hydrolysis followed by separation of insoluble residual compounds by centrifugation and concentration. The product pH was 4.4. The product electrical conductivity (EC) increases linearly with increasing PH concentration (C) in the water with the following relationship between EC (dS m^−1^) and C (ml L^−1^) in pure water:

$$\text{EC}=0.1983\;\text{C}\;({\text{R}}^{2}=0.996)$$

PH biostimulant Trainer^®^ contains 5% of N (w/w) as free amino acids, and soluble peptides. The aminogram of the product was (%): alanine—Ala (1.2), arginine—Arg (1.8), aspartic acid—Asp (3.4), cysteine—Cys (0.3), glutamic acid—Glu (5.4), glycine—Gly (1.2), histidine—His (0.8), isoleucine—Ile (1.3), leucine—Leu (2.2), lysine—Lys (1.8), methionine—Met (0.4), phenylalanine—Phe (1.5), proline—Pro (1.5), threonine—Thr (1.1), tryptophan—Trp (0.3), tyrosine—Tyr (1.1), valine—Val (1.4) (Paul et al., 2019b). The macronutrient composition of Trainer^®^ is as follows (%): P (0.09), K (0.41), Ca (0.07), and Mg (0.1). Trainer^®^ also contains the following micronutrients (mg kg^−1^): Fe (30.0), Mn (1.0), B (1.0), Zn (9.6), and Cu (9.0).

Ultrafiltration was carried out using a molecular weight cut-off (MWCO) cellulose acetate membrane (cellulose acetate, VWR, Milan, Italy) of 0.5–1 and 8–10 kDa, following manufacturer recommendations. The two MWCO were chosen to target the fractionation between small molecules and oligopeptides (around 9 amino acid residues—MWCO 0.5–1 kDa), and polypeptides (up to 90 amino acid residues—MWCO 8–10 kDa), respectively. Water was used for partition, and the product was allowed to diffuse for 24 h. At the end of partition, total N was measured in each fraction for both MWCO, through the Dumas' method using an elemental analyzer (Elemental vario MAX CN, Langenselbold, Germany). The nitrogen analysis showed low concentrations of N in all the PH-fractions in comparison to the whole product, due to the dilution that took place in the fractionation process. The total N content of each fraction was as follow (% w/w): 0.105 for PH1 (< 0.5–1 kDa); 0.861 for PH2 (> 0.5–1 kDa); 0.128 for PH3 (< 8–10 kDa); 0.384 for PH4 (> 8–10 kDa).

### *In Vivo* Bioassays

Tomato rooting test bioassay was carried out to identify the auxin-like activity by estimating the ability of the whole product and its fractions to promote initiation of adventitious roots in tomato cuttings (Matsumiya and Kubo, 2011; Colla et al., 2014). Tomato (*Solanum lycopersicum* L. cv. Marmande, SAIS Sementi, Cesena, Italy) seeds were surface sterilized using commercial bleach with sodium hypochlorite at 2% for 20 min. After being raised with sterilized water, the tomato seeds were sown in a germination tray filled with vermiculite. The growth chamber had a 12 h photoperiod with a light intensity of 450 µmol m^−2^ s, air temperature of 24°C, and 65% relative humidity. After 25 d from sowing, 3-true leaf tomato cuttings were harvested. In the experiment 1, cuttings were immersed for 5 min in a solution (basal application) containing three rates of either the PH Trainer^®^ or its fractions whereas distilled water and indole-3-butyric acid (IBA) were used as negative and positive control, respectively. Since the amino acids and peptides are organic nitrogenous compounds, a normalization of application rates for PH and its fractions was carried out in order to apply the same level of nitrogen for each dose level in experiment 1 (3, 30, or 300 mg L^−1^ of N). Product rates at each nitrogen level changed as follow (g L^−1^ for 3, 30, or 300 mg L^−1^ of N, respectively): PH1 (2.86, 28.57, 285.71), PH2 (0.35, 3.48, 34.84), PH3 (2.34, 23.44, 234.38), PH4 (0.78, 7.81, 78.12), and PH (0.06, 0.60, 6.00). IBA was applied at three rates as follow: 0.006, 0.06, and 0.6 g L^−1^. In experiment 2, the following treatments were tested: water-treated control, foliar application of a water solution containing PH at 6 g L^−1^, basal application of a water solution containing PH1 at 285.71 g L^−1^, foliar application of a water solution containing PH1 at 285.71 g L^−1^, and basal application of a solution containing IBA at 0.06 g L^−1^. PH1 was selected as the most active fraction based on Experiment 1. In all treatments, the applied rate of PH or its fraction (PH1) was established in order to assure the same level of N (300 mg L^−1^ of N). Foliar application of PH or its fraction (PH1) was made by a quick dip of aerial part of cuttings into the solution. The cuttings of both experiments were planted in transparent plexiglas boxes containing 8 cm of wetted perlite. The boxes were closed to ensure a relative humidity close to saturation (100%). Treatments were arranged in a randomized complete block design with three replications. Each experimental unit consist of a box containing 30 cuttings. After 7 d from planting, the roots of cuttings were gently washed with distilled water, until the root systems were free from any perlite particles. The measurement of the total root length was made on 18 cuttings per treatment using a WinRHIZO Pro (Regent Instruments Inc., Canada), connected to a STD4800 scanner. In the second experiment additional 18 cuttings per treatment (6 cuttings per experimental unit) were sampled for metabolomics analysis. With this latter aim, cuttings were removed and gently washed with distilled water, the basal part was sampled (2.5 cm of basal part of cutting stems including roots) and immediately quenched by dipping in liquid nitrogen, then stored at −80°C until analyses.

### Metabolomics

Samples of tomato cuttings gained from the *in vitro* assays were grinded with liquid nitrogen using pestle and mortar, and then extracted as previously reported (Rouphael et al., 2018a). Briefly, an aliquot (1.0 g) was extracted in 10 ml of 0.1% HCOOH in 80% aqueous methanol using an Ultra-Turrax (Ika T-25, Staufen, Germany). The extracts were centrifuged (12,000 × *g*) and metabolomic analysis was then carried out by UHPLC liquid chromatography quadrupole-time-of-flight mass spectrometry (UHPLC/QTOF-MS) as previously reported (Pretali et al., 2016). With this aim, a 1290 ultra-high-performance liquid chromatograph, a JetStream electrospray ionization source and a G6550 QTOF (all from Agilent technologies, Santa Clara, CA, USA) were used. Reverse phase chromatography was carried out on an Agilent Zorbax Eclipse-plus C18 column (100 × 2.1 mm, 1.8 μm) using a linear binary gradient elution (5%–95% methanol in water in 34 min, flow rate 220 μl/min). The mass spectrometer was operated in SCAN mode (100–1000 m/z) and positive polarity.

Mass (5 ppm difference in accuracy) and retention time (0.05 min as maximum shift allowed) alignment, as well as a filter-by-frequency post-processing were done in post-acquisition using Agilent Profinder B.06 software. For filtering purposes, only the compounds annotated in at least 75% of replications within at least one treatment were retained. The combination of monoisotopic mass, isotopes ratio and spacing was used to annotate compounds. The reference database was PlantCyc 12.6 (Plant Metabolic Network, http://www.plantcyc.org; downloaded April 2018). According to COSMOS Metabolomics Standards Initiative (http://cosmos-fp7.eu/msi), our identification corresponded to Level 2 (putatively annotated compounds).

### Statistical Analysis

In both bioassay experiments 1 and 2, ANOVA tests were conducted using the software package SPSS 10 for Windows (SAS Inc., Cary, NC). Duncan's multiple range test was performed at *p* = 0.05 on each of the significant variables measured.

Chemometric interpretation of the metabolomics dataset was carried out using Mass Profiler Professional B.12.06, as previously described (Salehi et al., 2018). Compounds abundance was Log2 transformed, normalized at the 75^th^ percentile, and baseline against the median. Unsupervised hierarchical cluster analysis (HCA) was done to describe relatedness/distance of metabolomic signatures across treatments. With this aim, the heatmap based on fold-change values was used, similarity was set as “Euclidean” and the “Wards” linkage rule was chosen. The dataset was then loaded into SIMCA 13 (Umetrics, Malmo, Sweden), Pareto-scaled and Orthogonal Projections to Latent Structures Discriminant Analysis (OPLS-DA) supervised analysis was carried out. Outliers were preliminary investigated using Hotelling's T2 (95% and 99% confidence limits for suspect and strong outliers, respectively). CV-ANOVA (*p* < 0.01) and permutation testing (N = 300) were also applied to validate the model and to exclude overfitting. Goodness-of-fit R^2^Y and goodness-of-prediction Q^2^Y were calculated for the OPLS-DA model and finally, Variable Importance in Projection (VIP) analysis was used to select the most discriminant metabolites. The metabolites included in the dataset were subjected to fold-change analysis and ANOVA in a Volcano analysis, to describe the extent and direction of regulation following biostimulant treatments. Metabolites derived from Volcano analysis with their fold-change values were finally imported into PlantCyc pathway Tools software (Karp et al., 2010) to highlight the pathways and processes involved in plant response to treatments.

## Results

### Biostimulant Action of PH and Its Fractions on Tomato Rooting

**Figure 1:**
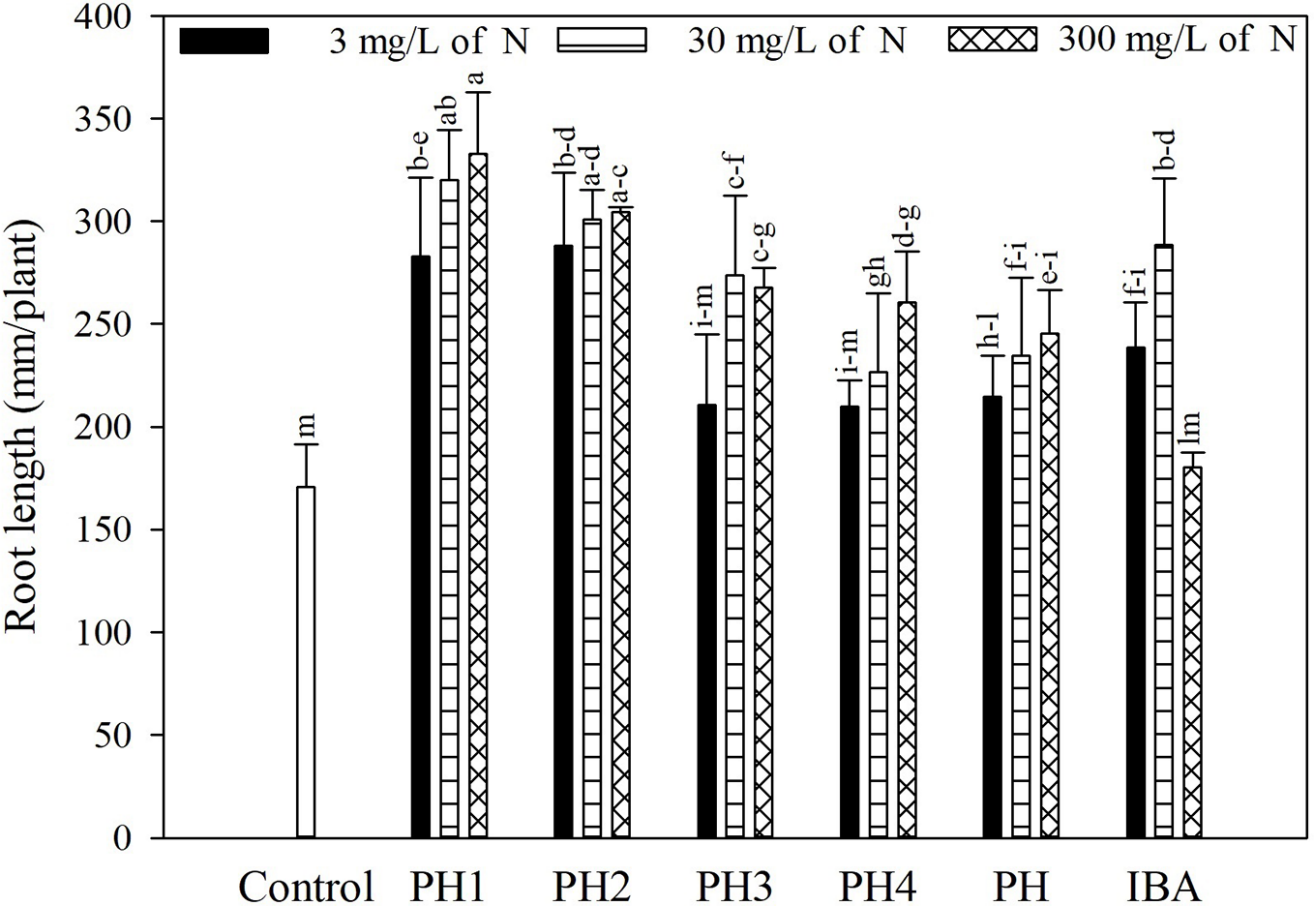
Root length of tomato plants as affected by basal treatment of cuttings with solutions containing the protein hydrolysate Trainer^®^ (PH) or one of its fractions (PH1 = fraction with molecular weight below 0.5–1 kDa; PH2 = fraction with molecular weight above 0.5–1 kDa; PH3 = fraction with molecular weight below 8–10 kDa; PH4 = fraction with molecular weight above 8–10 kDa), or indole-3-butyric acid (IBA) in the experiment 1. All products were applied at three doses. Different letters over bars indicate significant differences between treatments according to Duncan's multiple range test (*p* = 0.05).

**Figure 2:**
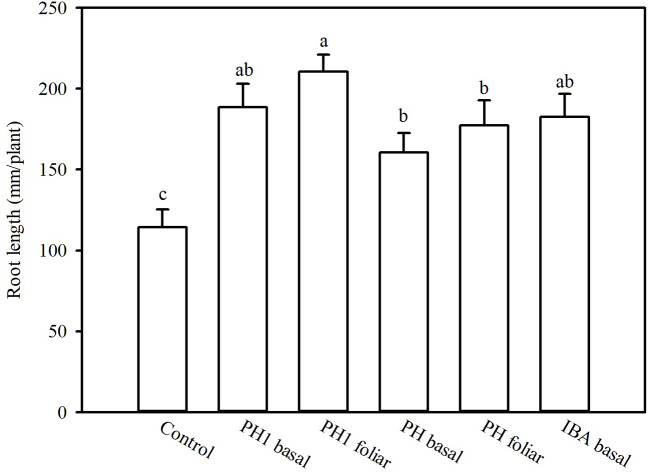
Root length of tomato plants as affected by basal or foliar treatment of cuttings as carried out in the experiment 2. To this aim, solutions containing distilled water, protein hydrolysate (PH) Trainer^®^ or its fraction with molecular weight below 0.5–1 kDa (PH1) or indole-3-butyric acid (IBA) have been tested. Different letters over bars indicate significant differences between treatments according to Duncan's multiple range test (*p* = 0.05).

Overall, both the PH and its fractions increased the root length, compared to control (experiment 1; **Figure 1**). The highest root length (342.8 mm/plant) was obtained in the treatment PH1, corresponding to the small fraction (MWCO < 0.5–1 kDa) at dose 3, whereas the lowest value was observed in control treatment (170.9 mm/plant; **Figure 1**). In the experiment 2, PH1 and IBA were the most efficient treatments in promoting root growth in comparison with negative control (+83 and 64% for PH1 foliar and basal application, respectively; + 59% for IBA treatment). PH-treated cuttings showed intermediate values of root length (**Figure 2**).

### Untargeted Metabolomics

A comprehensive analysis based on plant metabolomic profiling was performed. The untargeted metabolomic approach, using a hybrid quadrupole-time-of-flight mass spectrometer coupled to an UHPLC chromatographic system (UHPLC-ESI/QTOF-MS), was carried out to investigate the metabolic reprogramming induced by treatments and then to discern the pathways and processes elicitated by PH application.

**Figure 3:**
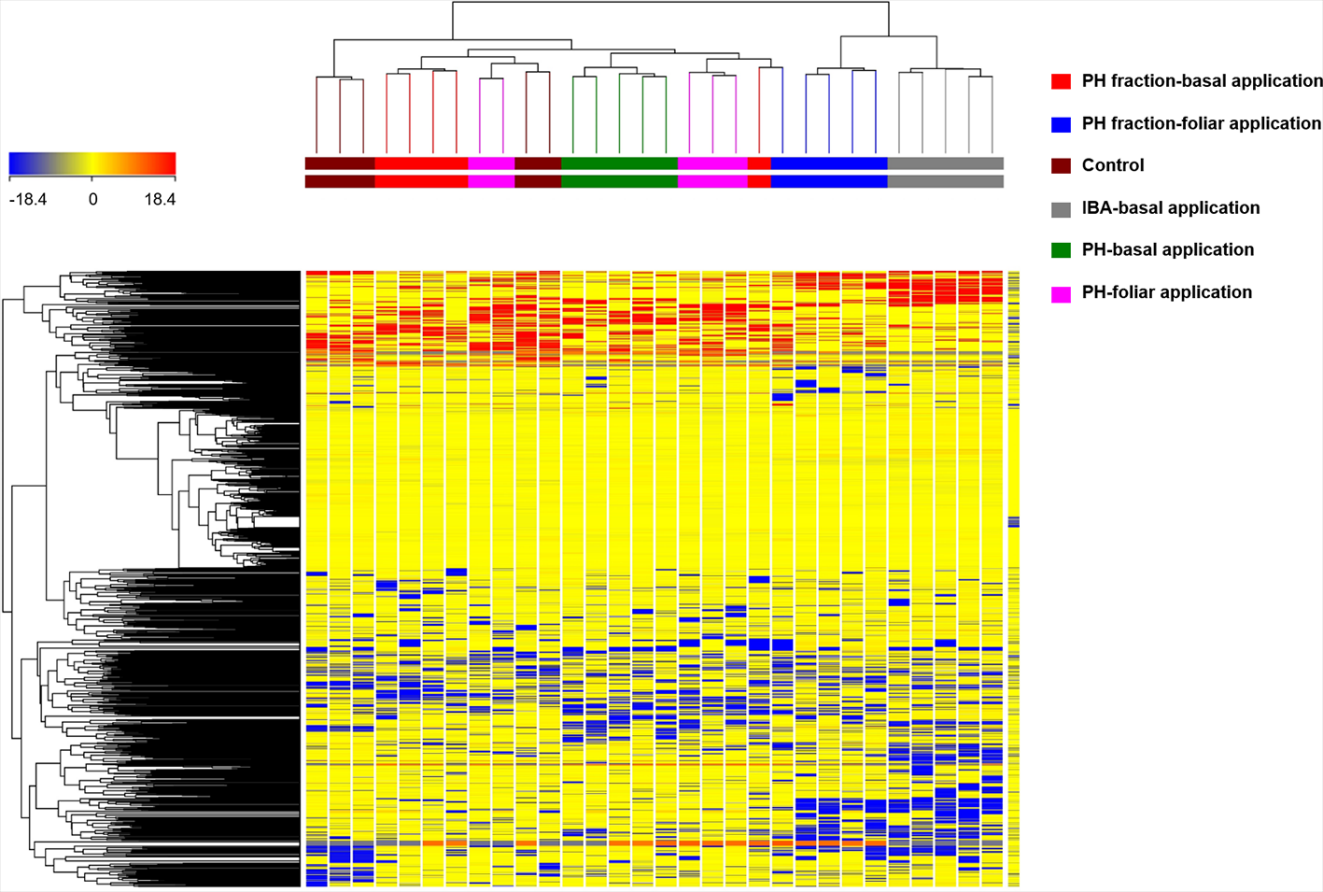
Unsupervised hierarchical cluster analysis carried out from UHPLC-ESI/QTOF-MS metabolomic analysis of tomato plants treated with a protein hydrolysate (PH) or its fraction PH1 (MWCO < 0.5–1 kDa) either *via* basal or foliar application (Exp. 2). Indole-butyric acid (IBA) and water were used as positive and negative control, respectively. The fold-change based heat map from compounds' normalized intensities was used to build hierarchical clusters (linkage rule: Ward; distance: Euclidean).

In a preliminary step, the HCA allowed to group the samples according to their similarities/dissimilarities in an unsupervised manner. The fold-change (FC) of metabolites provided by UHPLC-ESI/QTOF-MS analysis were used to build the HCA heat-map and the relative clusters, as a first step of interpretation. The complete list of metabolites annotated, with individual intensities, is provided as supplementary material (**Supplementary Table 1**). HCA outcome (**Figure 3**) indicated that the changes in the metabolic profile could differentiate the effect of the different treatment on tomato plants. The clustering results showed that the samples were grouped in two principal clusters. The first cluster grouped samples from IBA treatment (positive control) and PH1 with foliar application, thus indicating a close relationship between the two. The second cluster included a sub-cluster grouping the (not fractioned) PH and PH1 with basal application, as well as another sub-cluster including the replicates from negative control. Therefore, this unsupervised analysis suggested that the treatments induced a change in metabolomic profile of tomato, and that such changes resulted from the combination of the material considered (PH *vs* PH1) and the mode of application (basal *vs* foliar).

**Figure 4:**
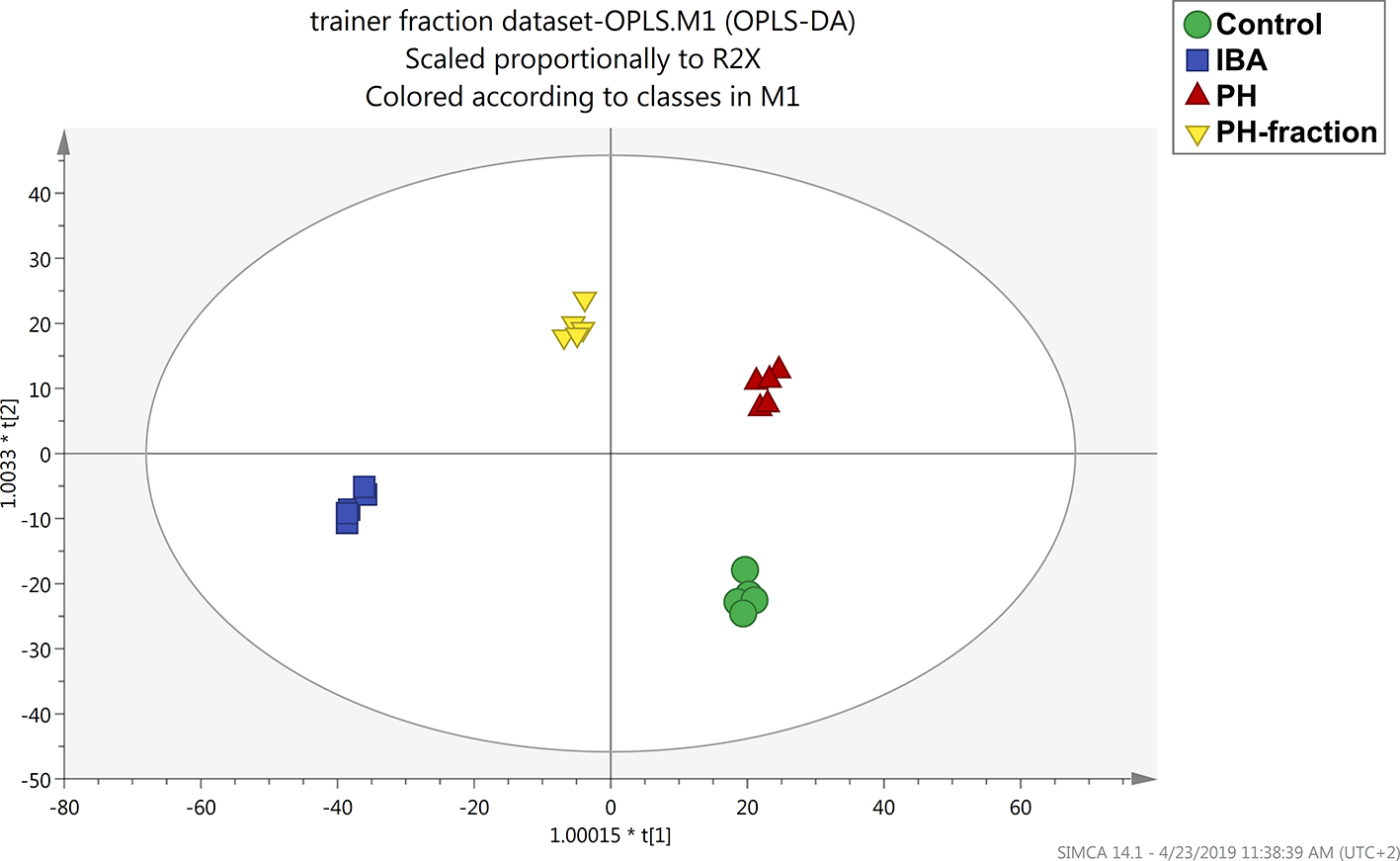
Orthogonal projection to latent structures discriminant analysis (OPLS-DA) supervised modeling of tomato plants following basal application of a protein hydrolysate (PH) or its PH1 fraction (MWCO < 0.51 kDa) (Exp 2). The metabolomic dataset produced through UHPLC-ESI/QTOF-MS was Pareto scaled and then used for the multivariate OPLS-DA modeling. Indole-butyric acid (IBA) and water were used as positive and negative control, respectively.

To better understand the effect of each biostimulant and the differences between them, a supervised multivariate analysis was next performed considering the basal application of PH and PH1 together with the two controls (water and IBA). OPLS discriminant analysis allowed to effectively separate the treatments into the score plot hyperspace, by discerning predictive and orthogonal components of variance (**Figure 4**). All the treatment resulted well separated from each other, suggesting a different effect at molecular level. The model was validated, and the parameters indicated a good predictivity (R2Y=0.989; Q2Y= 0.8; CV-ANOVA *P* = 4.28E^−7^). At the same time, permutation testing excluded model overfitting.

VIP analysis was therefore used to identify the metabolites mostly involved in the separation between treatments. Metabolites presenting a VIP score >1.4 were considered as discriminant and used for the discussion. These discriminant compounds identified by the supervised approach are provided in **Supplementary Table 2**. Among these metabolites, secondary metabolites were the most represented compounds suggesting that the treatment had a specific effect on the secondary pathways. In fact, hormones such as gibberellins and brassinosteroids were the principal discriminants isoprenoids. In addition, we underlined the presence of alkaloids and some glucosinolates, together with polyphenols as isoflavones and lignans.

A comprehensive overview of the metabolic processes involved in tomato plant response to the treatments was provided by the PlantCyc software. With this aim, the most significant compounds obtained from Volcano analysis (P-value<0.05; FC>1.5) were loaded into the Pathway Tools Omics Dashboard. This tool allowed to interpret the changes at molecular level and to link to a putative physiological process and, therefore, to expand the knowledge regarding the mode of action of the bioactive component(s) of the PH.

**Figure 5:**
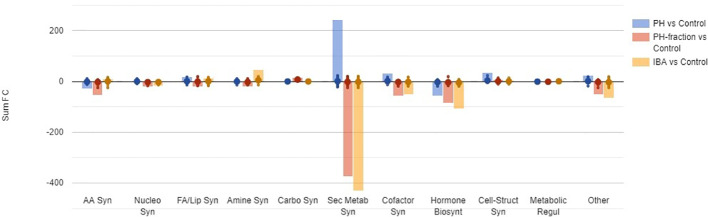
A summary of biosynthesis processes involved in tomato plant response to foliar application of a protein hydrolysate (PH) or its PH1 fraction (MWCO < 0.5–1 kDa) (Exp. 2). The metabolomic dataset produced through UHPLC-ESI/QTOF-MS was subjected to volcano plot analysis (P<0.05, fold-change > 1.5) and differential metabolites loaded into PlantCyc Pathway Tool (https://www.plantcyc.org/). Indole-butyric acid (IBA) and water were used as positive and negative control, respectively. The x-axis represents each set of subcategories while the y-axis corresponds to the cumulative fold-change. AA Syn: amino acids biosynthesis; Nucleo Syn: nucleoside and nucleotide biosynthesis; FA/Lip Syn: fatty acid and lipid biosynthesis; Amine Syn: amine and polyamine biosynthesis; Carbo Syn: carbohydrate biosynthesis; Sec Metab Syn: secondary metabolite biosynthesis; Cofactor Syn: cofactor, prosthetic group, electron carrier, and vitamin biosynthesis; Hormone Biosynt: hormone biosynthesis; Cell-Struct Syn: cell structure biosynthesis; Metabolic Regul: metabolic regulator biosynthesis; Other: other biosynthesis.

**Figure 5** summarizes the differential metabolites, classified by categories based on their role in the biosynthesis pathways. As a broad overview, PH-fraction 1 showed a similar profile to positive control IBA, and different from PH. Secondary metabolism included the most intensely modulated categories of compounds, in response to PH and its fraction application (**Table 1**). Indeed, over 250 compounds included in secondary metabolism related pathways were affected by the treatments. Similarly, hormones and compounds belonging to cofactors, prosthetic groups, electron carrier's biosynthesis, and vitamins were identified as a general plant response to treatments. However, the intensity of the metabolite modulation and the carbon and nitrogen fluxes appeared to be distinct, based on the treatment. In this sense, PH induced an up accumulation in secondary metabolism and cofactors-related compounds, and a down accumulation of hormones. However, the fraction PH1 showed a behavior differing from the PH, that resulted in a down accumulation of several secondary metabolism biosynthetic pathways, in line with IBA treatment.

**Table 1:** Summarized biosynthesis processes involved in plant response to foliar application of a protein hydrolysate (PH) or its fraction PH1 (MWCO < 0.5–1 kDa) (Expt. 2).

	Log FC PH			Log FC PH-fraction			Log FC IBA		
	No. compounds	Average	Sum FC	No. compounds	Average	Sum FC	No. compounds	Average	Sum FC
**Amino acid biosynthesis**	25	1.2	−29.9	25	−2.1	−53.7	25	0.4	9.2
**Nucleosides and nucleotides biosynthesis**	8	0.3	2.2	8	−2.6	−20.8	8	−2.3	−18.7
**Fatty acid and lipid biosynthesis**	24	0.8	18.9	24	−0.9	−21.7	24	0.5	11.7
**Amines and polyamines biosynthesis**	10	−0.4	−4.4	10	−2.2	−22.0	10	4.5	45.3
**Carbohydrates biosynthesis**	1	−0.1	−0.1	1	14.7	14.7	1	0.2	0.2
**Secondary metabolites biosynthesis**	252	0.9	221.2	254	−1.4	−344.1	252	−1.7	−425.1
**Cofactors, prosthetic groups, electron carriers biosynthesis**	27	1.3	33.8	27	−2.1	−57.0	27	−1.9	−52.3
**Hormones biosynthesis**	25	−2.3	−58.0	25	−3.4	−85.7	25	−4.3	−107.6
**Cell structures biosynthesis**	11	3.3	36.0	11	0.7	7.7	11	0.4	4.1
**Metabolic regulators biosynthesis**	3	−0.8	−2.3	3	−1.3	−3.9	3	0.5	1.5
**Other biosynthesis**	27	0.7	18.0	27	−3.1	−82.7	27	−3.1	−83.8

The metabolomic dataset produced through UHPLC-ESI/QTOF-MS was subjected to volcano plot analysis (P < 0.05, fold-change > 1.5) and differential metabolites loaded into PlantCyc Pathway Tool (https://www.plantcyc.org/). Indole-butyric acid (IBA) and water were used as positive and negative control, respectively. The average and summed Log fold-changes (Log FC) values, together with the number of compounds involved, is provided for each biosynthetic pathway and for each treatment.

The biochemical processes including nitrogen-containing secondary metabolites, phenylpropanoids and terpenes were the most elicited by the biostimulant treatments, as confirmed from volcano plot (p< 0.05, FC > 1.5) analysis for differential metabolites (**Supplementary Table 3**). Flavonoids and alkaloids and, to a lesser degree glucosinolates, were particularly up-accumulated in PH treated-plants. It is worthy to note that compounds as N-feruloyltyramine, intermediate in the biosynthesis of suberin, and some lignans as (+)-secoisolariciresinol monoglucoside and (−)-pluviatolide, were up-accumulated in the presence of PH. The defence compounds indole-3-carboxylate and psoralen, both considered phytoalexins, were found to be stimulated by PH.

On the contrary, the fraction PH1 showed a different effect on these compounds, since a general response to the treatment depressed the biosynthesis related to secondary metabolism and phytoalexins plant defence. Phenylpropanoids, predominantly flavonoids, terpenophenolics, as well as terpenoids, polyketides, and alkaloids were negatively affected by PH1 application. Despite the general decrease of defence compounds, metabolites as the fucocumarin psoralen or the phenylpropanoid ferulate were up accumulated. As recorded following PH application, the alkaloid isoalliin were highly increased. Interestingly, anthranilate, intermediate in the pathway of tryptophan and its related pathways, and the 3-hydroxycinnamic acid were down-accumulated in both PH-fraction 1 and IBA. The phytosiderophores mugineate and 3-epihydroxy-2'-deoxymugineate presented the same down-accumulation trend in both fraction PH1 and IBA.

Notably, also phytohormones presented a modulation as a consequence of the treatments (**Supplementary Figure 1**). Brassinosteroids, cytokinins, and jasmonates biosynthesis related compounds were down accumulated following application of PH. The pattern of the phytohormones in response to fraction PH1 and IBA application was very similar. Abscisic acid related compounds and cytokinins were down accumulated in the presence of both fraction PH1 and IBA. However, gibberellins were elicited by all treatments.

## Discussion

PHs are considered as plant biostimulants since they can have a positive effect on plants by increasing shoot and root biomass, rather than tolerance to abiotic stresses, thus promoting crop productivity (Colla et al., 2015). This positive effect could be linked to the interference of PH on nutrient uptake and Fe, C, and N metabolism (Colla et al., 2015; Paul et al., 2019a). The above findings can also be linked to the increased availability of nutrients for plant uptake resulting form the formation of metal complexes of amino acids and peptides in PHs. Similarly, humic substances improvement of plant iron nutrition as a consequence of metal complexation by humic substances (HS) extracted from different sources has been widely reported. In our study, OPLS-DA supervised multivariate modeling and the following VIP analysis indicated differences in the secondary metabolism and suggested that each treatment (i.e. the PH rather than its fraction) had a specific effect at metabolome level. The most active fraction of the PH (PH1, MWCO <0.5–1 kDa) was that including oligopeptides and amino acids. Santi et al. (2017) observed that PHs containing peptides and a low quantity of free amino acids presented a higher effect on root growth and micronutrient accumulation than free amino acid mixture. Similarly to HS (Zanin et al., 2019), the improvement of micronutrient uptake resulting from the PH applications can be related to the direct effects of PH on micronutrient-acquisition mechanisms and to the capability of amino acids and peptides to form stable complexes with metals. Moreover, small peptides have been postulated as key signaling molecules, since they could regulate various aspects of developmental processes in plants (Oh et al., 2018).

A general accumulation of nitrogen-containing compound was showed after PH application, in agreement with a previous study revealing that the PH enhances nitrogen uptake (Colla et al., 2014). In addition, probably due to the modulation of nitrogen metabolism, alkaloids presented a variation of their amount. The modulation of alkaloids is consistent with previous findings using a PH on lettuce (Lucini et al., 2015); these compounds are one of the largest groups of plant secondary metabolites containing nitrogen in their structure. Among other, the complex and partially understood roles ascribed to alkaloids in plant metabolism included the regulation of plant growth and the action as reservoir of nitrogen (Waller and Nowacki, 1978; Facchini, 2001). Several studies linked the nitrogen content and the bioavailability of N to the increase of alkaloids (Sreevalli et al., 2004; Banani et al., 2017). Moreover, it has been postulated that the increase of N assimilation due to the PH application could stimulate phenylpropanoids pathway (Colla et al., 2015), which could explain the changes observed in the phenylpropanoid metabolites following PH application. Noteworthy, several works reported that biostimulants are effective in modulating the profile of phenolic compounds in wine (Boselli et al., 2019; Salvi et al., 2019), tomato (Lucini et al., 2015), and pepper (Barrajón-Catalán et al., 2020). In plant, phenolics play a plethora of functions both in terms of physiology and development as well as regarding interactions with biotic and abiotic environments.

Besides the modulation of secondary metabolism, that was expected, it is important to point out that the PH and even more its fraction PH1 strongly affected the profile of phytohormones. Interestingly, PH1-induced metabolomic changes showed always the same trend as IBA-induced changes. This is coherent with the outcome of *in vivo* bioassays, where this fraction showed the highest auxin-like activity, similarly to IBA. The effect of the not fractionated PH was distinct from PH1, thereby indicating that fractionation enriched the product by the component having an auxin-like activity that promoted the growth of adventitious roots. The fractionation-related activity of biostimulant materials has been previously described for HS (Maggioni et al., 1987; Puglisi et al., 2009), even though the knowledge about the underlying mechanisms still remains partial and fragmentary (Calderin Garcia et al., 2016). With this regard Nardi et al. (2002) highlighted that the portions with low molecular weight (<3,500 Da) HS could easily reach the plasmalemma of plants, whereas high molecular weight fractions (>3,500 Da) could interact only with the cell wall. Similarly, Canellas et al. (2010) observed different hormone-like activities when testing different size fractions of a vermicompost humic substance. Although referring to a different biostimulant material, these different clues contribute to support the distinctive differences in metabolic signatures we observed between PH and its low molecular weight fraction PH1.

This latter PH1 fraction provided an auxin-like activity when applied to tomato. It is known that auxins play an important role in plant development, including rooting (Enders and Strader, 2015). Root growth is sustained by the apical meristem, a region near the root tip where the development program regulates cell division and elongation and is pivotally maintained by auxins, cytokinins, and gibberellins. In particular, auxins play a key role in root development, being involved in the positioning and formation of the meristem, and stimulation of mitotic activity (Muraro et al., 2016). In fact, auxins are reported to promote founder cells of both shoots and roots (Toyokura et al., 2019). Nevertheless, it is important to keep in mind that root development is the result of a rather complicated and still poorly understood coordinated multilayer interaction network between auxins, cytokinins, gibberellins, and ethylene (Liu et al., 2017). Cytokinins regulate the activity of the meristem antagonistically to auxins, negatively modulating the transport of auxins (Marhavý et al., 2011) and reducing mitotic activity *via* the promotion of cell differentiation (Dello Ioio et al., 2007). As far as gibberellins are considered, their involvement in root development is important during the early stages when they promote auxin transport and cell proliferation (Moubayidin et al., 2010). The hormonal regulation of root growth can be further expanded, since the gibberellin-related DELLA proteins are known to interact with jasmonate, ethylene, and brassinosteroids, (Liu et al., 2017), the latter acting antagonistically to auxins by inhibiting cell elongation in the root tip (Chaiwanon and Wang, 2015). Finally, abscisic acid coordinates auxins to determine root elongation and architecture, even under no stress conditions (Harris, 2015).

Notably, this complex regulation network can be linked to our results. The biostimulant fraction PH1 (*i.e.*, the fraction inducing the same metabolic modulation caused by IBA), determined a coordinated hormonal change that supports the auxin-like activity observed in the bioassays. The application of PH1 caused a down accumulation of cytokinins (antagonists of auxins in root development), and a concurrent down accumulation of abscisic acid intermediates (inhibitor of root elongation). The increase in gibberellins observed following application of both PH and PH1, can have promoted auxins transport and cell proliferation while inhibiting brassinosteroids, these latter being antagonists of the auxins. Noteworthy, the biostimulants-induces increase in gibberellins has been previously linked to rooting of azalea cuttings, especially at the early stage of root development (Elmongy et al., 2018). Furthermore, the whole metabolite signature (mainly targeting secondary metabolism) was largely shared between IBA- and PH1-induced metabolomic reprogramming, thereby corroborating the auxin-like activity resulting from the changes in phytohormone profiles. Besides, we cannot exclude a direct contribution of the endogenously present auxin in determining to the shift in metabolic patterns we observed following application of PH1. The long-distance effects we observed in tomato plants are hard to be substantiated without specific labeling experiments. Bearing in mind that auxins are known to be transported from the shoot to the root apex (Muraro et al., 2016), we can speculate that this process might have supported the increased rooting when PH1 was foliarly applied. On the other hand, the ability of biostimulants to affect tissues other than those of application has been demonstrated by Kulikova et al. (2016) using a tritium-labeled humic acid.

It must be noted that also the non-fractionated PH modulated the hormone profile in tomato, with brassinosteroids, cytokinins, and jasmonates being down accumulated and gibberellins being accumulated following the treatment (**Figure 5**). Noteworthy, these changes are only partially shared with PH1 and to some extent are specifically induced by this treatment. This finding is in agreement with the fact that PH1 is a fraction of PH, where some components are enriched (small molecules, oligopeptides) and some other are depleted (higher molecular weight compounds).

In general, regardless the molecular mechanisms involved, small peptides have been confirmed as potentially important signaling compounds, as previously postulated for the product used in this work (Colla et al., 2014). Indeed, they could regulate various aspects of developmental processes in plants (Oh et al., 2018). Concerning root development, peptide hormones have been proposed as a key mechanism for cell–cell interactions in plants (Yamada and Sawa, 2013). These signaling peptides coordinate both development and responses to environment (Toyokura et al., 2019); despite their mechanism of action in the shoot is well known, their role in the root is relatively uncharacterized (Yamada and Sawa, 2013).

## Conclusions

A combination of molecular fractionation by dialysis, *in vivo* bioassay for hormone-like activity, and metabolomics, has been successfully tested to identify the most active fraction of a vegetal derived-PH. Auxin-like activity was tested through a rooting assay, to preliminary screen the PH and its fractions, in comparison to the auxin IBA (positive control). This rooting experiment allowed identifying the fraction (PH1) having the highest auxin-like activity, to be tested further, in combination with MS-based metabolomics, to shed light onto the biochemical processes underlying the activity observed. The combination of fractionation (effective in reducing the complexity of a chemically diverse matrix like PH) with metabolomics was effective to depict the changes induced by the tested fraction at biochemical level. Such complementary contributions are effective to investigate the possible mode of action and the most bioactive components of a biostimulant product.

The smallest fraction of PH containing small molecules and oligopeptides (molecular weight < 1 kDa) was the most active in promoting the rooting of tomato cuttings. Moreover, metabolomics allowed to identify the mode of action of PH and its fraction (PH1) in comparison with the exogenously applied IBA. PH1 and IBA-treated cuttings showed a similar metabolomic signature (mainly affecting secondary metabolism and phytohormone profiles), thereby corroborating the auxin-like activity. Notably, *in vivo* bioassays were consistent with metabolomics, considering that PH1 was actually effective in promoting the growth of adventitious roots.

Therefore, provided that our work focused on auxin-like activity, PH1 was identified as the most active fraction of the PH. The results suggested that this approach is suitable to understand the biological activity retained by the different fractions in a complex biostimulant product. Noteworthy, the unfractionated PH was not devoid of biostimulant properties, suggesting that the choice of the best fraction(s) depends on the desired activity. Although the present work focused on auxin-like activity, it worth to consider that the fractions other than PH1 might be of interest, when different aims are to be targeted. The comprehension of active fractions can assist the manufacture of more effective biostimulants, where the yield of the desired fraction/s is optimized during the production process. With this regard, the present approach represents a good solution that can be applied in all cases where biostimulants are based on complex mixtures of bioactive substances differing in molecular weight, like for HS, PH, and/or algal extracts. Finally, future studies should also address the role of mineral nutrients in the biostimulant activity of PHs.

## Data Availability Statement

All datasets generated for this study are included in the article/**Supplementary Material**.

## Author Contributions

LL and BM-M performed the molecular fractionation and metabolomic study, data analysis and interpretation, and wrote many parts of the manuscript. YR, and MC performed the bioassays, data analysis and interpretation, and wrote many parts of the manuscript. GC and LL coordinated the whole project, provided the intellectual input, set up the experiments, contributed to the interpretation of the results, and corrected the manuscript.

## Conflict of Interest

The authors declare that the research was conducted in the absence of any commercial or financial relationships that could be construed as a potential conflict of interest.

## Supplementary Material

Supplementary Figure 1 Phytohormone biosynthesis processes involved in plant response to foliar application of a protein hydrolysate (PH) or its fraction (MWCO < 0.5–1 kDa) (Exp. 2). The metabolomic dataset produced through UHPLC-ESI/QTOF-MS was subjected to volcano plot analysis (P<0.05, fold-change > 1.5) and differential metabolites loaded into PlantCyc Pathway Tool (https://www.plantcyc.org/). Indole-butyric acid (IBA) and water were used as positive and negative control, respectively. The x-axis represents each set of subcategories while the y-axis corresponds to the cumulative fold-change.

Supplementary Table 1 Whole dataset produced from untargeted metabolomics carried out in tomato cuttings treated with either a protein hydrolysate (PH) or its fraction PH1 (Exp. 2). Compounds are presented with individual intensities, annotations and composite mass spectra (mass abundance combinations).

Supplementary Table 2 VIP discriminant compounds as identified from OPLS-DA discriminant analysis of tomato cutting metabolomic profiles following either a protein hydrolysate (PH), PH1 or indole-3-butyric acid application (Exp. 2).

Supplementary Table 3 Differential metabolites as derived from Volcano analysis (P-value<0.05, Bonferroni multiple testing correction; FC>1.5) in tomato cutting metabolomic profiles following either a protein hydrolysate (PH), PH1 or indole-3-butyric acid application (Exp. 2).
