# Supplementary figures and images for: Combining Molecular Weight Fractionation and Metabolomics to Elucidate the Bioactivity of Vegetal Protein Hydrolysates in Tomato Plants
Source: Front Plant Sci. 2020 Jun 30;11:976. doi: 10.3389/fpls.2020.00976 (PMC7338714; doi:10.3389/fpls.2020.00976)

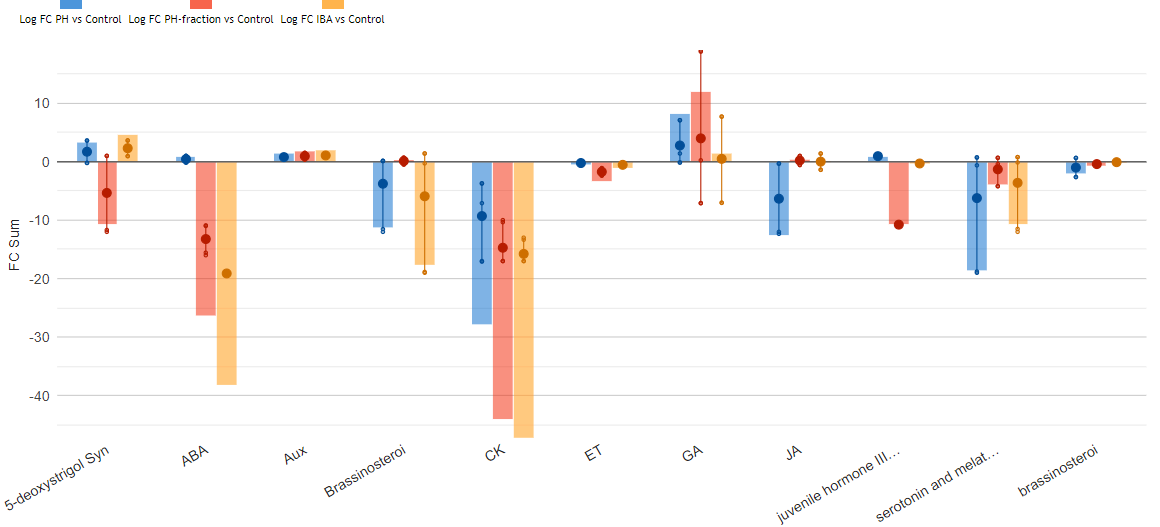

Supplement: Supplementary Figure 1 — Phytohormone biosynthesis processes involved in plant response to foliar application of a protein hydrolysate (PH) or its fraction (MWCO < 0.5–1 kDa) (Exp. 2). The metabolomic dataset produced through UHPLC-ESI/QTOF-MS was subjected to volcano plot analysis (P<0.05, fold-change > 1.5) and differential metabolites loaded into PlantCyc Pathway Tool (https://www.plantcyc.org/). Indole-butyric acid (IBA) and water were used as positive and negative control, respectively. The x-axis represents each set of subcategories while the y-axis corresponds to the cumulative fold-change. [file Image_1.tif]
